# Effectiveness of violet LED with or without a bleaching gel: a 12-month randomized clinical trial

**DOI:** 10.3389/fdmed.2024.1427301

**Published:** 2024-10-15

**Authors:** Ana Cristina Távora de Albuquerque Lopes, Nair Cristina Margarido Brondino, Juliana Fraga Soares Bombonatti, Rafael Francisco Lia Mondelli

**Affiliations:** ^1^ Department of Operative Dentistry, Endodontics and Dental Materials, Bauru School of Dentistry, University of São Paulo (USP), São Paulo, Brazil; ^2^ Department of Mathematics, São Paulo State University (UNESP), São Paulo, Brazil

**Keywords:** in-office tooth bleaching, violet LED, dental esthetic, teeth sensitivity, tooth whitening

## Abstract

**Introduction:**

The present interventional, controlled, randomized, blind clinical study aimed to evaluate the effectiveness of an in-office bleaching procedure with violet LED associated or not with 37% carbamide peroxide, considering as response variables the degree of change and color stability over 12 months and dental sensitivity over a month.

**Methods:**

Forty participants, according to the inclusion and exclusion criteria, were randomly divided into 2 groups (*n* = 20) according to the bleaching protocol conducted, in two sessions, with a 7-day interval: vLED—violet LED, without gel; vLED/CP—37% carbamide peroxide photocatalyzed with violet LED (control group). In the vLED group, in each session the bleaching was carried out by 2 consecutive irradiation cycles of 25’ each (10 × 2’ LED + 30” interval), with 5’ interval between cycles. In the vLED/CP group, the gel was applied 5 times in the bleaching session and photocatalyzed 3 times for 2’ with 30” intervals (7’30” per gel application), totaling 37’30” per session. Dental sensitivity was assessed using a visual analog scale (VAS) and the effectiveness of bleaching as a function of the degree of change and color stability (*Δ*E) with a spectrophotometer. The data were tabulated and submitted to statistical tests (*p* < 0.05).

**Results:**

The VAS analysis showed that some individuals from both groups had mild pain (1 ≤ VAS < 4) during the time intervals evaluated, being more prevalent in the vLED/CP group. Regarding the degree of color change, the groups behaved differently over time (*p* < 0.0001). The *Δ*E observed for the vLED/CP group was superior in comparison to the vLED group at all evaluated moments.

**Conclusions:**

Over 12 months, the vLED/CP group was more effective in relation to the bleaching effect compared to the vLED group. Both groups showed low levels of sensitivity in the studied time intervals.

**Clinical Trial Registration:**

[https://ensaiosclinicos.gov.br/rg/RBR-6rc23h], identifier [U1111-1253-8850].

## 1 Introduction

Dental esthetics is an important factor in the composition of the current beauty standard recommended by society (1). Both a smile and teeth appearance are related to facial attractiveness and contribute to consequences on self-esteem, social interaction, and psychological health (2). Whiter teeth are often desired; hence, tooth bleaching is a procedure performed routinely in dental offices. The advantages of bleaching encompass being a simple low-invasive procedure with interesting cost-benefits and satisfactory results (1).

Several energy sources (halogen light, plasma arc, LED, LED-laser, and laser) have been associated with bleaching gels to promote thermocatalysis of peroxide. The reactive oxygen species interact with the pigmented organic macromolecules, causing them to break down into smaller, colorless molecules. Consequently, it is expected to reduce the clinical operative time in tooth bleaching procedures in which a light source is used (1, 3–4), in addition to reducing tooth sensitivity (3). However, the benefit of using light sources is still controversial and frequently discussed (7, 8).

The most frequent side effect after the bleaching treatment is dental sensitivity, which can be attributed to the presence of oxygen bubbles inside the dentinal tubules after the application of the bleaching gel with the peroxide penetrating the pulp, causing irritation and the risk of irreversible pulpitis (4, 5, 9, 10). Another side effect caused by bleaching gels, in function of pH levels, is the possibility to decrease the enamel microhardness, increasing the enamel surface roughness and wear (11–14). To control these side effects in office dental bleaching, some authors employed a low concentration of hydrogen peroxide or carbamide peroxide gels photoactivated with a violet LED light source (15–20), avoiding an increase in the pulper chamber temperature during the treatment (21). Thus, the development of bleaching protocols without gel would probably contribute to a lower incidence of sensitivity and could be promising. Although considered subjective, the most used method to assess tooth sensitivity has been the VAS scale (Visual Analogue Scale) (3, 5, 6, 15, 17).

New bleaching products and light sources have been continuously introduced in the dental market. A violet LED system, with a wavelength ranging from 405 to 410 nm, has been presented to promote the breaking of chromophore macromolecules through a physical process (1, 15, 17, 18). *In vitro* and *in vivo* studies have shown that using the violet LED alone can produce enough energy to promote the breakdown of pigments in tooth enamel (15, 17, 18, 22–27).

The present clinical study aimed to investigate the effectiveness of bleaching vital teeth (in-office) with violet LED associated or not with 37% carbamide peroxide gel, the degree of sensitivity over 1 month, and the color stability over 12 months. The null hypotheses evaluated were: (1) The use of violet LED without bleaching gel will not be effective in in-office bleaching; (2) The use of violet LED associated with the bleaching gel based on carbamide peroxide will not be effective in in-office bleaching; (3) The use of violet LED with and without bleaching gel will not provide dental sensitivity after bleaching.

## 2 Material and methods

### 2.1 Study design

The present interventional, controlled, randomized, blind clinical study compared 2-session bleaching protocols with violet LED associated or not with 37% carbamide peroxide. The response variables were the degree of change and color stability over 12 months and dental sensitivity over 1 month. The description of the experimental design follows the guidelines of the Consolidated Standards of Reporting Trials (CONSORT) and was approved by the Local Research Ethics Committee (Protocol: 2.731.030/CAAE: 90570218.4.0000.5417; Bauru School of Dentistry, University of São Paulo, Brazil).

### 2.2 Randomization and allocation

The calculations to determine the sample size were performed using the GLIMMPSE program (http://glimmpse.samplesizeshop.org/), as described in Guo & Pandis (2015) and were based on the values of *ΔE*. We calculated the sample size for a repeated measures design, considering one between-subjects effect (treatment) and one within-subjects effect (moment). Additionally, we used Huynh-Feldt corrections. The power was set at 80%, the significance level at *α* = 0.05, and effect sizes were estimated based on the mean results from Delafiori (28). To model the covariance, we assumed a standard deviation of 1.3 for each outcome and used an unstructured covariance matrix. We obtained group sizes of 13 and 7 to test the main effects of time and treatment, respectively. Considering a 25% dropout rate, the sample size was set at 16 participants per group. After receiving approval from the Research Ethics Committee, participants were recruited from undergraduate and graduate students. Forty participants, aged 18–35 years, were selected based on the inclusion and exclusion criteria (Table 1).

**Table 1:** Inclusion and exclusion criteria.

Inclusion criteria
• Sign the consent form; • Availability to attend all sessions; • Age between 18 and 35 years; • Good health, controlled blood pressure, and adequate oral hygiene; • Pulp vitality in the teeth to be bleached (1st PM on one side to 1st PM on the opposite side in the upper and lower arches); • Color of teeth above A2 on the VITA scale visually identified.
Exclusion criteria
• Smoker; • Pregnant or lactating; • Heart problems; • History of known reaction to peroxides; • Individual or family history of neoplasia in the oropharynx region and surroundings; • History of diabetes or other systemic diseases which can interfere with the access of tissues from the oral cavity; • Need antibiotic therapy before dental prophylaxis; • Presence of oral pathologies, xerostomia, caries lesions, extensive restorations of composite resin, fractures or splinters in the teeth, gingivitis/periodontitis, bruxism, which in the professional's opinion may compromise the participant's health or the results of the study; • Presence of surface irregularities, tetracycline stain, discoloration due to trauma, fluorosis, hypoplasia, dental implant, prosthesis and/or endodontic treatment in the upper or lower anterior teeth, or other parameters that may make it difficult to measure tooth color; • Have made use of bleaching agents in the dental office or at home in the last year (does not include toothpaste or bleaching mouthwash); • Spontaneous tooth sensitivity; • Intention to put a fixed orthodontic appliance during the bleaching assessment period.

Participants were informed about the risks and benefits of participating in the research and signed an informed consent form. They were then numbered from 1 to 40 and randomly assigned to two groups (*n* = 20) using the RANDBETWEEN formula in Microsoft Excel (Microsoft, Redmond, WA, USA). The groups were based on the bleaching protocol used: vLED group, which underwent violet LED bleaching without gel, and the vLED/CP group, which received 37% carbamide peroxide gel photocatalyzed with a violet LED (control group).

### 2.3 Intervention: bleaching procedures

Each participant received complete supragingival prophylaxis performed with a rubber cup, pumice, and water. A clinical mirror was used for an intra-oral examination of the anterior teeth. The presence of composite or remaining resin restorations, after removal of the orthodontic brackets, was verified using optical fluorescence equipment (Evince, MMOpitcs Ltda., São Carlos, SP, Brazil).

Any excess of composite resin on the buccal surface of the anterior teeth was removed with multi-laminated drills and sanding discs for polishing composite resin.

Next, a photographic documentation was obtained. Initial tooth color was measured objectively using a spectrophotometer (Vita Easyshade Advance 4.0, VITA Zahnfabrik H. Rauter GmbH & Co. KG, Bad Säckingen, Germany) and dental sensitivity was measured using an analog visual scale (VAS).

In the vLED/CP group, the gingival tissues around the teeth on the buccal surface were protected with a gingival barrier (TOP DAM, FGM, Joinville, SC, Brazil), while in the vLED group, no gingival barrier was used. Two bleaching sessions were performed for each participant according to the protocol defined for each group (Table 2). The bleached teeth ranged from the 1st premolar on one side to the 1st premolar on the opposite side, in the upper and lower arch, totaling 16 teeth per individual.

**Table 2:** Groups and treatments.

Groups	Light source	Bleaching gel	Bleaching protocols
vLED	Violet LED	–	Each session: Two light application cycles, with 25’ duration (10 × 2’ LED + 30” interval) each cycle and 5’ interval between them
vLED/CP	Violet LED	37% Carbamide Peroxide (Power Bleaching, BM4, Maringá, PR, Brazil)	Each session: Five applications of 37% PC activated with violet LED, following the photoactivation protocol 3 applications of light for 2’ with 30” intervals between them (7'30” per application of gel), totaling 37’30”

In both groups, the Bright Max Whitening device was used. It is composed of 4 violet LEDs (425 mW/cm^2^ each emitter, total optical power of 1.7 W) with a wavelength of 400 ± 10 nm (MMOptics Ltda., São Carlos, SP, Brazil).

The bleached teeth of the vLED group received neither polishing nor application of desensitizer. The bleached teeth of the vLED/CP group received, after bleaching, polishing on the buccal surface with a felt disc and polishing paste based on aluminum oxide (Ox Gloss 2, KG Sorensen, Cotia, SP, Brazil) and application of the desensitizer (Desensibilize KF 2%, FGM Ltda., Joinville, SC, Brazil) for 4’ in each bleaching session.

Participants were instructed not to ingest substances that could pigment their teeth in the first 48 h after the bleaching treatment. Such substances would include coffee, black tea, grape juice, red wine, soy sauce, tomato sauce, berries, açaí, mustard, ketchup, Coca-Cola, etc. In addition, they were also instructed not to use red lipsticks (women), not to smoke, and not to eat acidic food such as lemon juice, pineapple, orange, and soft drinks to avoid potentiating a possible initial demineralization of the tooth enamel caused by the bleaching agent.

### 2.4 Clinical assessment: color measurement

The photographic documentation of the participants was carried out with a Canon 80D digital camera. Digital photographs of the smile, intraoral, right, and left side were obtained with each color measurement. All research was carried out at the CRC (Clinical Research Center), Bauru School of Dentistry, University of São Paulo-USP, Brazil in the same clinic environment. The CRC features fluorescent lighting which provides control of the luminosity for taking all photographs and reading the color of the patients’ teeth, in complete control in this aspect.

The reference color was obtained initially (baseline) in the first session. The degree of maintenance and color change was measured 24 h after the 1st session, at the beginning of the 2nd session, 24 h, 07 days, 1 month, 6 months, and 12 months after the 2nd bleaching session. Color measurements were made with the Vita Easyshade® Advance 4.0 spectrophotometer (Vita-Zanhnfabrik, Bad Säckingen, Germany).

The spectrophotometer was calibrated before measurements according to the manufacturer's guidance. After initial prophylaxis, two consecutive measurements were carried out for each tooth on the middle third of the buccal surface. The recording was carried out when the values obtained were equal. If there was a discrepancy between the values obtained in the two readings, additional readings were carried out until two equal and consecutive readings were obtained. Only one measurement was recorded for each dental element at each time interval (3, 5, 6, 29). Measurements were always performed in an environment lit by a fluorescent lamp and by the same blind examiner who did not participate in the execution of the bleaching protocol.

All bleached teeth were evaluated, totaling 16 teeth per participant. The color was provided based on the CIELab color system. L* represents the brightness values, a* the red-green values, and b* the yellow-blue values. The numerical values measured for L*, a* and, b* were recorded and tabulated to obtain *Δ*E, using the formula $\Delta E=\sqrt{(\Delta a^{*}{)}^{2}+(\Delta b^{*}{)}^{2}+(\Delta L^{*}{)}^{2}}$ and subsequent statistical analysis (30).

### 2.5 Clinical assessment: dental sensitivity

A 10 cm VAS scale was used to assess tooth sensitivity before and after tooth bleaching (3, 5, 6, 15, 17). The participant recorded on a horizontal line with a 0–10 mark any tooth sensitivity that occurred. A vertical risk corresponded to the sensitivity level of the teeth, which could vary from no sensitivity (zero) to extreme sensitivity (ten). Records were carried out in the 1st session: before (initial) bleaching, immediately after, and 24 h after; and in the 2nd session: before bleaching, immediately after, 24 h, 7 days, and 1 month after. To avoid any bias, the patients were oriented to mark the level of sensitivity without any influence from the operator and they are left alone to mark the moments that were evaluated on the VAS scale.

### 2.6 Statistical analysis

To study the color variation (*Δ*E), all analyses were performed using the R software, version 3.6.0. The *lme4* (31), *plotly* (32) and *lmerTest* (33) libraries were also used.

To model the behavior of *Δ*E, we used a Linear Mixed Model with a random effect for teeth nested within the patient. The assumptions of normality and homoscedasticity of the residuals were verified using a histogram with a density normal curve and a residual vs. predicted values plot, respectively. Different models were compared using the likelihood ratio test (*p* < 0.05), the Akaike information criterion (AIC), and the Bayesian information criterion (BIC). Descriptive analysis was used to assess sensitivity. For pairwise comparisons, we used the Sidak test.

## 3 Results

**Figure 1:**
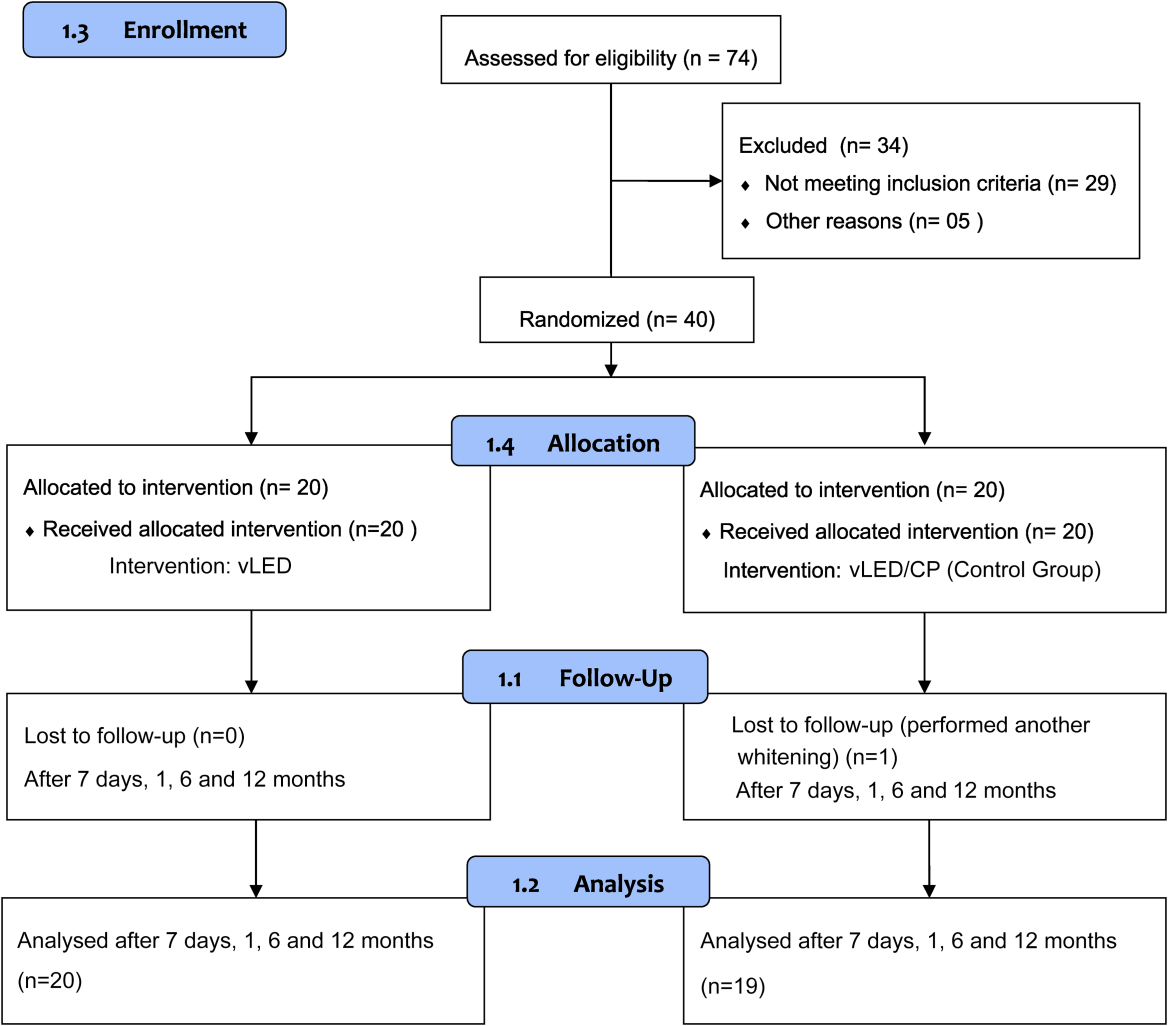
CONSORT flow diagram of the participants’ progress through the trial phases.

A total of 40 participants were included in the present research according to the inclusion and exclusion criteria and were followed up for 12 months (Table 1). A CONSORT flow diagram of the participants’ progress through the trial phases is depicted in Figure 1. The percentage of patients who were lost in the follow-up after 12 months was 2.5%. One individual patient did not attend the final 12-month assessment of the control group (vLED/CP).

The values of the simple means and standard deviations of each group, in the studied intervals, are shown in Table 3.

**Table 3:** Mean and standard deviation of the *Δ*E of the groups studied over 12 months.

Groups	24 h after the 1st session	Beginning of the 2nd session	24 h after the 2nd session	7 days after the 2nd session	1 month after the 2nd session	6 months after the 2nd session	12 months after the 2nd session
vLED	2.46 ± 0.45AB	2.42 ± 0.46AB	2.43 ± 0.44AB	2.29 ± 0.45A	2.59 ± 0.63AB	2.31 ± 0.41A	2.66 ± 0.81B
vLED/CP	3.76 ± 0.78C	3.90 ± 1.14C	5.05 ± 1.33DE	5.20 ± 1.34E	5.08 ± 1.33DE	4.75 ± 1.32D	4.05 ± 1.18C

Values for the groups at the times evaluated in different superscript letters significantly differ from each other (*p* < 0.05).

**Figure 2:**
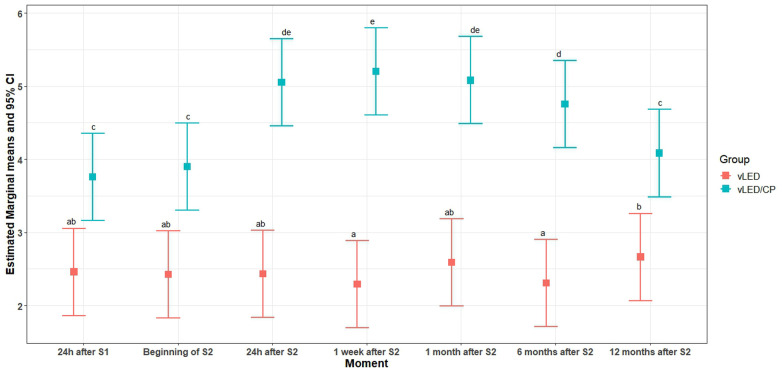
Behavior of *Δ*E groups studied.

Regarding the degree of color change, the graphical analysis of the residuals suggests no violation of the assumptions of normality and homoscedasticity. The likelihood ratio test suggested the inclusion of an interaction effect of moment × treatment (*p* < 0.0001) in the model. This conclusion is corroborated by the F test from ANOVA (*p* < 0.0001). The AIC and BIC criteria suggested the inclusion of a random effect for teeth nested within the patient. Figure 2 shows the estimated marginal means and 95% CI (confidence interval) for *Δ*E. The expected mean of *Δ*E observed for the vLED/CP group was always higher than that observed for the vLED group (*p* < 0.0001 in all comparisons). For the vLED group, the Sidak test rejected the null hypothesis of equality of means for the moments 12 months after the 2nd session and 1 week after the 2nd session (*p* = 0.007) and 6 and 12 months after the 2nd session (*p* = 0.01). For the vLED/CP group, the mean of *Δ*E observed 24 h after the 1st session was smaller than those observed at 24 h, 1 week, 1 month, and 6 months after the 2nd session (*p* < 0.0001 in all comparisons). The expected mean of *Δ*E observed 1 week after the 2nd session was greater than those observed 6 months after the 2nd session (*p* = 0.02) and 12 months after the 2nd session (*p* < 0.0001). Six months after the 2nd session, the expected mean of *Δ*E was statistically equal to those observed at 24 h (*p* = 0.2) and 1 month after the 2nd session (*p* = 0.1) for this group.

**Figure 3:**
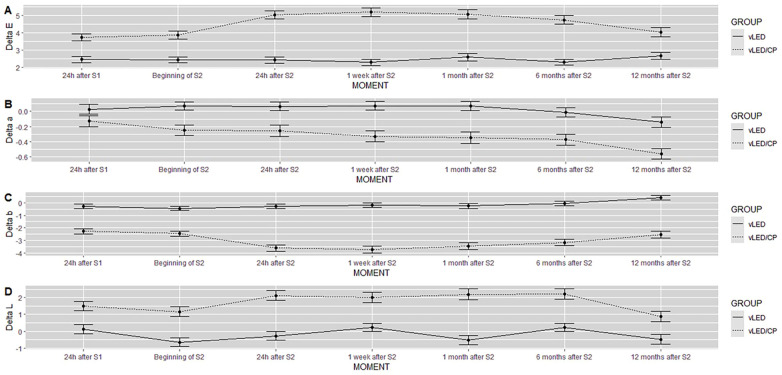
Graphs of Delta E **(A)**, Delta a **(B)**, Delta b **(C)** and Delta L **(D)** of the results to the groups evaluated.

Figure 3 shows the results of *Δ*L, *Δ*a, *Δ*b and *Δ*E for the control group (vLED/CP group) in comparison to the vLED group, always presented higher *Δ*L and *Δ*E in all moments. In contrast, the *Δ*a and *Δ*b values for the control group showed lower values at all times compared to the test group (vLED group), demonstrating the best whitening results for the control group.

**Figure 4:**
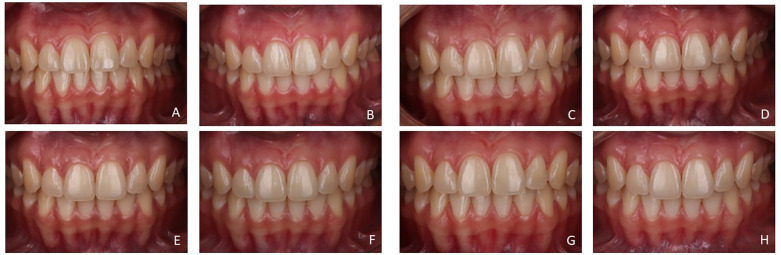
Participant of the vLED group in the time intervals: **(A)** beginning of the 1st session; **(B)** 24 h after the 1st session; **(C)** beginning of the 2nd session; **(D)** 24 h after the 2nd session; **(E)** 7 days after the 2nd session; **(F)** 1 month after the 2nd session; **(G)** 6 months after the 2nd session; **(H)** 1 year after the 2nd session.

**Figure 5:**
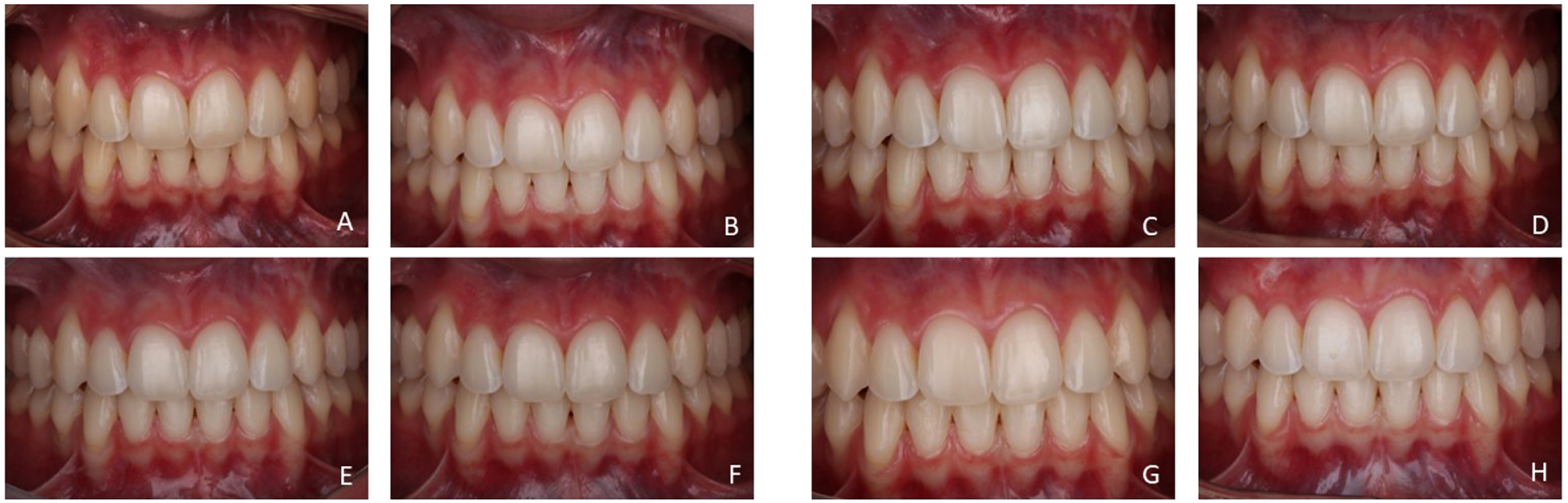
Participant of the vLED/CP group in the time intervals: **(A)** beginning of the 1st session; **(B)** 24 h after the 1st session; **(C)** beginning of the 2nd session; **(D)** 24 h after the 2nd session; **(E)** 7 days after the 2nd session; **(F)** 1 month after the 2nd session; **(G)** 6 months after the 2nd session; **(H)** 1 year after the 2nd session.

Figures 4A–H illustrates the case of a patient in the vLED group and Figures 5A–H illustrates the case of a patient in the vLED/CP group in the 8 periods assessed.

For the analysis of the VAS scale, pain levels were grouped according to the following criteria: VAS < 1—No pain; 1 ≤ VAS < 4—Mild pain; 4 ≤ VAS ≤ 7—Moderate pain; VAS > 7—Severe pain. It was assumed that at the initial moment all patients had no pain (initial VAS equal to zero). Table 4 shows the counts of types of pain according to the group.

**Table 4:** Number of patients who presented sensitivity in the intervals evaluated.

Intervals	vLED group			vLED/CP group		
	No pain	Mild pain	Moderate pain	No pain	Mild pain	Moderate pain
Immediately after the 1st session	14	6	0	12	7	1
24 h after the 1st session	20	0	0	16	4	0
Beginning of the 2st session	19	1	0	17	3	0
Immediately after the 2st session	19	1	0	12	8	0
24 h after the 2nd session	19	1	0	15	5	0
7 days after the 2nd session	19	1	0	19	1	0
30 days after the 2nd session	20	0	0	19	1	0

## 4 Discussion

The present study aimed to verify the lightening efficiency, the dental sensitivity, and the color stability over 12 months in in-office bleaching procedures utilizing violet LED associated or not with 37% carbamide peroxide bleaching gel. The three null hypotheses were rejected. Both protocols studied were able to produce tooth bleaching with low sensitivity. The groups behaved differently over time (*p* < 0.0001). The vLED/CP group (control group) showed higher values of *Δ*E and *Δ*L than the vLED group in all periods and a higher incidence of sensitivity, although, in general, both protocols had a low degree of sensitivity (Figures 2, 3; Tables 3, 4).

When two objects are placed side by side under controlled conditions, the smallest color difference detected by the human eye is the value of *Δ*E equal to 1 (34). In the present study, vLED without gel promoted tooth bleaching, but to a lesser extent compared to the vLED/CP group (Figures 2–5; Table 3). After 12 months, the *Δ*E value of the vLED group without gel was 2.66 ± 0.81, which is also the highest value observed in this group among all intervals. The highest *Δ*E value observed in the vLED/CP group was 5.20 ± 1.34 (control group), corresponding to the period of 7 days after the second bleaching session. Twelve months after the second session, the *Δ*E value in the vLED/CP group was 4.05 ± 1.18 (Table 3). In general, there was a greater perception of dental whitening by patients in the vLED/CP group when compared to the vLED group, especially after the second whitening session. This factor did not allow for a longer evaluation time, as individuals in the vLED group were not satisfied with the result after 12 months and were seeking new whitening treatment.

De Souza Rastelli et al. (19) postulated that violet LED light alone could excite the organic compounds adhered to the enamel surface and break them down into smaller compounds. This phenomenon occurs because violet LED light emits photons that propagate at a shorter wavelength and a higher vibrational frequency than blue LED light. Consequently, it has physical properties characterized by low penetration into dental tissues and greater energy in surfaces (1, 15, 17, 18, 35). Thus, the mechanism of action of the violet LED light may perhaps be restricted to extrinsic pigments on the enamel surface (19, 36).

Pigmented macromolecules have chemical bonds that make electrons dislocated and very susceptible to light absorption with short wavelengths, such as that of violet LED light (37). Hence, the wavelength range emitted by the violet LED coincides with the absorption peak of the pigmented molecules. When absorbing the light, the molecules are excited and the chemical bonds can change from a situation of a strong union to one of a weak union, or the bonds may even break, making the molecules smaller (38–40). If the fragments do not recombine, the molecule stops absorbing and the color center disappears, i.e., the structure is cleared. This process also occurs in almost all objects with pigmented molecules, such as fabrics and plastics, for example (18).

Carbamide peroxide dissociates into hydrogen peroxide and urea. Urea dissociates in water and ammonia; this reaction raises the pH of the solution and contributes to reducing enamel demineralization. The proteolytic activity of urea may also increase the effectiveness of bleaching due to the greater release of the free radical peridroxil (40, 41).

Gallinari et al. (38) evaluated bleaching of bovine teeth in a study that included 3 sessions with 35% or 17.5% hydrogen peroxide, photoactivated or not with a violet LED light source. Researchers found that the use of violet LED alone cannot replace traditional treatment; however, it can produce favorable results when associated with low concentrations of peroxide, such as 17.5% hydrogen peroxide, for example. In the present study, the association of violet LED with 37% carbamide peroxide (control group) provided greater indices of delta L and delta E, with a decrease of the tooth pigments (*Δ*a and *Δ*b, Figure 3), determining better results for the control group in relation to the test group.

In an *in vitro* research with bovine teeth in which 37% carbamide peroxide was associated with violet LED, Kury et al. (42) observed that violet light significantly increased the effectiveness of carbamide peroxide even after 14 days of bleaching. Since carbamide peroxide has a low rate of decomposition, violet light may have accelerated the breakdown of peroxide by increasing the temperature.

In bleaching with 10% carbamide peroxide associated or not with the violet LED, Gallinari et al. (39) observed that both groups had chromatic saturation 14 days after treatment. However, the hemi-arch that received irradiation with violet LED light showed more chromatic changes than the non-irradiated side. Thus, it was suggested that in addition to the oxidation of organic pigments by reactive oxygen species promoted by the bleaching gel, the violet LED must have acted on pigments difficult to be oxidized by peroxide, leading to greater changes in *Δ*E on the irradiated side.

In the present study, the second bleaching session for the vLED/CP group (control group) contributed to increasing the chromatic change, which in turn, remained relatively stable until 6 months after bleaching and then reduced. However, in the group whose bleaching was performed with vLED, without gel, the second bleaching session did not contribute significantly to greater bleaching efficacy, i.e., saturation occurred already in the first bleaching session. In addition, the color change obtained in the first session in the group without gel remained relatively stable after 12 months of postoperative control. Thus, the bleaching protocol carried out only with vLED reached its best result in the first session and remained more stable over time, therefore, it was more predictable than when combining 37% carbamide peroxide (Figure 2; Table 3).

The manufacturer of the violet LED light source employed in the present research warns that the response of each patient to bleaching with a violet LED light is very particular. Bleaching is intense in some patients after a single application, whereas, in other patients, even after several attempts, the result is small due to individual variations of the dental structure, which are beyond the professional's control. The limitations of bleaching with a violet LED without gel are related to the lack of stability of the breaks that occurred in the connections of the chromogenic macromolecules; consequently, unwanted re-pigmentation can take place (18).

Regarding sensitivity, although it is the most common side effect in tooth bleaching, even when moderate to severe pain is present, it tends to reduce or disappear within 24 h after the procedure (3, 5, 6, 41). However, the literature also records longer periods of postoperative sensitivity (40). In any case, it is known that sensitivity varies significantly from person to person (43).

In the present study, among the patients who had sensitivity in both groups, the pain intensity was mild (1 ≤ VAS < 4), except for one patient from the vLED/CP group, who presented moderate intensity (4 ≤ VAS ≤ 7) immediately after the first bleaching session. Thus, both protocols studied produced a low incidence of sensitivity (Table 4).

In bleaching performed only with a violet LED light source without gel, there is no chemical reaction. The process of breaking the pigmented molecules is carried out more selectively with minimal interaction with the dental structure as a whole (18, 38–40). Violet LED also acts on an electromagnetic spectrum capable of biologically interacting without causing molecular damage. Consequently, there is less risk of side effects such as sensitivity and enamel alterations (14, 18).

Dental enamel acts as a semipermeable membrane through which water and molecules with low molecular weight, such as oxygen ions released by peroxides, can diffuse (5). Light sources, in turn, are utilized on tooth bleaching based on the hypothesis that the light emitted on the bleaching gel is absorbed and partially converted into heat, thus increasing the rate of release of reactive oxygen species and the efficiency of bleaching. Thus, the light acts as a catalyst for the degradation of the bleaching gel to facilitate its diffusion into the dental structure (3, 5, 21, 38).

However, reactive oxygen species can not only oxidize pigmented agents but also spread to the pulp chamber and stimulate an inflammatory reaction that may cause morphological changes and decrease the rate of mitochondrial respiration in MDPC-23 odontoblastic cells as observed *in vitro* (44).

Afferent nociceptors express receptors that can be chemically activated and are involved in the pain mechanism, among which is tartrate-resistant acid phosphatase—1 (TRAP-1). Peroxides and other oxidizing agents oxidize cysteine residues in the TRAP-1 channel, activating it. In addition, the intracellular reaction of peroxide with Fe2 + produces free radicals (OH) via the Fenton reaction and also contributes to the activation of TRAP-1 (41, 44). Experiments in rodents have shown that TRAP-1 is the ion channel responsible for pain induced by oxidizing agents. This is an important mechanism for the spread of injury caused by free radicals in biological systems (44). The development of tooth bleaching protocols capable of reducing or eliminating the diffusion of peroxide to the pulp is therefore interesting.

A recent study (45) showed that gels with higher viscosity promoted a lower concentration of peroxide in the pulp chamber than gels with low and medium viscosity. The carbamide peroxide used in the present research is a viscous gel and, consequently, it may have contributed to a lower amount of peroxide reaching the pulp chamber and a lower incidence of sensitivity. Due to its low penetrability, the violet LED light also promotes more superficial heating of the dental elements (21) and, consequently, causes less molecular alteration in the deeper dental tissues, in addition to preserving the pulp from possible damage (17, 18, 46).

It is known that pain is an individual experience that is diverse and influenced by various factors such as personality, memories of past experiences of pain, emotion, and culture. The quality of pain is usually expressed in words. Although widely used, the VAS analysis is one method that has the limitation of contemplating only the aspect of pain intensity (47). Thus, the mild sensitivity reported by one of the participants in the vLED/CP group, even after 30 days of the bleaching procedure, may be attributed to individual pain perception factors (Table 4).

From the results obtained in the present research, the violet LED has shown the potential to clinically promote tooth bleaching. When associated with 37% carbamide peroxide (vLED/CP), better results are obtained than using vLED without gel. The use of 37% carbamide peroxide is interesting because it releases a low concentration of hydrogen peroxide which tends to promote less occurrence and intensity of dental sensitivity (15). Thus, the use of violet LED associated with a low concentration gel can be recommended for patients with a history of dental sensitivity. The stability over 12 months of the color obtained with the vLED/CP protocol also proved to be satisfactory, corroborating the indication of this treatment in clinical practice, mainly for young patients or young adults.

Despite the possibility of using violet LED without gel to provide the desired whitening with minimal or no sensitivity, not all patients have a favorable response to this gel-free protocol, where the best results are obtained in patients with extrinsic pigmentation. Another possibility is to start without gel, only with violet LED, and later associate a low concentration whitening gel activated with violet LED.

However, there are patients with a significantly low pain threshold, to whom the minimum stimulus can promote severe sensitivity and even cause the choice to abandon treatment. Especially in these cases, bleaching with violet LED without gel can be a viable alternative to present these patients with a possibility to perform the tooth bleaching procedure. Other clinical situations in which the bleaching with violet LED without gel is especially indicated are for young patients with a wide pulp chamber; patients with erosion, abrasion and abfraction lesions, gingival retractions, enamel microfractures, and dentin expose by bruxism and/or restorations with extensive interface restorative/enamel material.

## 5 Conclusions

Within the limits of the present research, it can be concluded that:

- The vLED group without gel was able to promote tooth bleaching;
- The vLED/CP group was more effective in relation to the lightening effect over the evaluated periods compared to the vLED group;
- Both protocols produced low levels of dental sensitivity in the studied time intervals.

## Data Availability

The datasets presented in this study can be found in online repositories. The names of the repository/repositories and accession number(s) can be found in the article/Supplementary Material.
